# NEAT1 inhibits the angiogenic activity of cerebral arterial endothelial cells by inducing the M1 polarization of microglia through the AMPK signaling pathway

**DOI:** 10.1186/s11658-024-00579-5

**Published:** 2024-04-29

**Authors:** Ting Chen, Xin Huang, Yi-Xuan Zhao, Zhi-wen Zhou, Wen-sheng Zhou

**Affiliations:** Department of Neurology, Hunan Provincial People’s Hospital (The First Affiliated Hospital of Hunan Normal University), Changsha, 410005 China

**Keywords:** NEAT1, Microglia, Cerebrovascular endothelial cells, Angiogenesis, AMPK

## Abstract

**Background:**

Enhancing angiogenesis may be an effective strategy to promote functional recovery after ischemic stroke. Inflammation regulates angiogenesis. Microglia are crucial cells that initiate inflammatory responses after various brain injuries. Long noncoding RNA nuclear paraspeckle assembly transcript 1 (NEAT1) plays a role in regulating brain injury. This study aimed to explore the effects of NEAT1-regulated microglial polarization on the neovascularization capacity of cerebrovascular endothelial cells and the underlying molecular regulatory mechanisms.

**Methods:**

Mouse cerebral arterial endothelial cells (mCAECs) were co-cultured with BV-2 cells in different groups using a Transwell system. NEAT1 expression levels were measured by fluorescence quantitative reverse transcription PCR. Levels of IL-1β, IL-6, TNF-α, Arg-1, IL-4, and IL-10 were determined using ELISA. Expression levels of CD86 and CD163 were detected by immunofluorescence. The neovascularization capacity of mCAECs was assessed using CCK-8, Transwell, Transwell-matrigel, and tube formation assays. Label-free quantification proteomics was carried out to identify differentially expressed proteins. Protein levels were measured by Western blotting.

**Results:**

NEAT1 overexpression induced M1 polarization in BV-2 cells, whereas NEAT1 knockdown blocked lipopolysaccharide-induced M1 polarization in microglia. NEAT1-overexpressing BV-2 cells suppressed the angiogenic ability of mCAECs, and NEAT1-knocking BV-2 cells promoted the angiogenic ability of mCAECs under lipopolysaccharide treatment. Label-free quantitative proteomic analysis identified 144 upregulated and 131 downregulated proteins that were induced by NEAT1 overexpression. The AMP-activated protein kinase (AMPK) signaling pathway was enriched in the Kyoto Encyclopedia of Genes and Genomes analysis of the differentially expressed proteins. Further verification showed that NEAT1 inactivated the AMPK signaling pathway. Moreover, the AMPK activator 5-aminoimidazole-4-carboxamide ribonucleotide reversed the effect of NEAT1 on BV-2 polarization and the regulatory effect of NEAT1-overexpressing BV-2 cells on the angiogenic ability of mCAECs.

**Conclusions:**

NEAT1 inhibits the angiogenic activity of mCAECs by inducing M1 polarization of BV-2 cells through the AMPK signaling pathway. This study further clarified the impact and mechanism of NEAT1 on microglia and the angiogenic ability of cerebrovascular endothelial cells.

**Supplementary Information:**

The online version contains supplementary material available at 10.1186/s11658-024-00579-5.

## Background

Ischemic stroke is the second leading cause of disability and death worldwide, with the heaviest disease burden occurring in low- and middle-income countries [1]. Intravenous thrombolysis and mechanical thrombectomy are the only effective treatments for ischemic stroke [2, 3]. A new approach to promoting the physiological recovery of brain-injured areas is to repair the damaged vascular system in the brain [4]. Angiogenesis, the process of creating new small blood vessels from existing ones through the growth and movement of endothelial cells, can improve cerebral blood flow and aid in the recovery of brain function after ischemia [5]. Increased microvascular density in the perilesional area of the cerebral infarction is associated with longer survival in patients with ischemic stroke [5]. Enhancing angiogenesis may be a strategy to promote functional recovery after ischemic stroke.

Angiogenesis is regulated by inflammation [6]. Microglia are crucial cells that initiate inflammatory responses after various brain injuries [7, 8]. They are versatile cells that interact with numerous cells in the central nervous system, including neurons, astrocytes, and oligodendrocytes [7, 9]. Microglia comprise 10–15% of the cells in the central nervous system [10]. In response to various stresses, microglia swiftly activate and differentiate into M1 or M2 phenotypes, also referred to as microglial polarization [11, 12]. CD11b, CD16, CD32, and CD86 are the surface markers of M1 microglia, whereas CD163 and CD206 are the surface markers of M2 microglia [13]. M1 microglia release proinflammatory cytokines, such as interleukin (IL)-1β, IL-6, and tumor necrosis factor-α (TNF-α), and promote brain injury [13]. Conversely, M2 microglia aid in the repair of brain injuries by secreting anti-inflammatory cytokines such as arginase-1 (Arg-1), transforming growth factor-β, IL-4, IL-10, and neurotrophic factors [13]. Although several studies have reported a relationship between microglial polarization and angiogenesis [14, 15], the molecular mechanisms of microglial polarization involved in brain angiogenesis were not fully elucidated. Further research is required to enrich the current literature.

Long noncoding RNAs play a significant role in regulating microglial polarization [16]. The long noncoding RNA nuclear paraspeckle assembly transcript 1 (NEAT1) is closely associated with regulating neural cell differentiation, function maintenance, and apoptosis and participates in the regulatory processes of neural injury [17, 18]. NEAT1 directly binds to the potassium channel-interacting proteins potassium voltage-gated channel subfamily A regulatory beta subunit 2 and potassium voltage-gated channel interacting protein 1, contributing to the modification of neuronal stress responses and is involved in the regulation of cellular apoptosis and autophagy during ischemia/reperfusion injury, thus potentially serving as a novel target for treating ischemic stroke [19, 20]. NEAT1 is also involved in microglial polarization [21, 22]. However, the role of NEAT1-regulated microglial polarization in the damage and repair of brain tissue resulting from ischemic stroke was not fully revealed.

To further clarify the role of NEAT1 in ischemic stroke, we explored the effects of NEAT1-regulated microglial polarization on the neovascularization capacity of cerebrovascular endothelial cells and the underlying molecular regulatory mechanisms. This study aimed to provide new insights into targets for the prevention and treatment of ischemic stroke-induced brain injury.

## Materials and methods

### Cell culture

Mouse cerebral arterial endothelial cells (mCAECs, Cat NO.: CP-M100) and BV2 retroviral-immortalized microglia (CL-0493A) were obtained from Procell Life Science and Technology Co. Ltd. (Wuhan, China). BV-2 cells were cultured in Dulbecco’s modified Eagle’s medium supplemented with 10% fetal bovine serum (FBS), 2 mM l-glutamine, 100 mg/mL streptomycin, and 100 U/mL penicillin. The mCAECs were cultured in a specific culture medium provided by Procell Life Science & Technology Co., Ltd. All the cells were cultured in a CO_2_ incubator at 37 °C, 5% CO_2_, and saturated humidity.

### Construction of NEAT1 overexpression plasmid

The full-length coding sequence of mouse NEAT1 (NR_003513) was amplified using NEAT1-F (5′-cggggtaccGTAGGAGTTAGTGACAAGGAGGGCTCGCTCTT-3′, Kpn I) and NEAT1-R (5′-ataagaatgcggccgcTTTTTTTCTAAGAAGCTTCAATCTCAAAC-3′, Not I) primers. The PCR amplification products were digested and ligated into the pcDNA3.1 + vector. After digestion and sequencing verification, the NEAT1 overexpression plasmid was successfully obtained and named pcDNA-NEAT1.

### BV-2 cell group

To investigate the role and mechanism of NEAT1, BV-2 cells were classified into six groups. The first group, referred to as the blank group, consisted of BV-2 cells cultured under normal conditions without intervention. The second group, named the empty vector group, involved the transfection of BV-2 cells with the empty vector pcDNA3.1+. The third group, designated the pcDNA-NEAT1 group, was transfected with pcDNA-NEAT. In the fourth group, BV-2 cells were exposed to lipopolysaccharide (LPS; 1 μg/mL). The fifth group included BV-2 cells treated with 1 μg/mL LPS and simultaneously transfected with a negative control siRNA (siNC), labeled as the LPS + siNC group. The sixth group comprised BV-2 cells treated with 1 μg/mL LPS and transfected with siRNA targeting NEAT1 (siNEAT1), denoted as the LPS + siNEAT1 group.

To confirm whether NEAT1 regulates the angiogenic ability of mCAECs via the AMP-activated protein kinase (AMPK) pathway, BV-2 cells were classified into three groups. The first group, referred to as the empty vector + dimethyl sulfoxide (DMSO) group, consisted of BV-2 cells transfected with pcDNA3.1 + empty vector and treated with DMSO. In the second group, named the pcDNA-NEAT1 + DMSO group, BV-2 cells were transfected with pcDNA-NEAT1 and treated with DMSO. The third group, designated as the pcDNA-NEAT1 + 5-aminoimidazole-4-carboxamide ribonucleotide (AICAR) group, entailed the transfection of BV-2 cells with pcDNA-NEAT1 and treatment with the AMPK activator AICAR.

All groups were transfected using Lipofectamine® 3000 (Invitrogen, Carlsbad, CA, USA). The transfection procedure adhered strictly to the manufacturer’s instructions.

### Fluorescence quantitative reverse transcription polymerase chain reaction (PCR)

Total RNA from the six groups of BV-2 cells was isolated using TRIzol reagent (Invitrogen). After quality assessment, total RNA was reverse-transcribed into cDNA. Next, a 20-µL PCR reaction mixture was prepared using cDNA as the template, following the instructions provided by YEASEN Biotechnology Co., Ltd. (Shanghai) for the SYBR Green qPCR Mix. The prepared PCR mixture was loaded into an ABI PRISM^®^ 7500 Sequence Detection System (Foster City, CA, USA) for quantitative PCR analysis. The relative expression levels of the target gene NEAT1 were determined using the 2^(-Delta Delta C(T)) method. The primer sequences (5′–3′) for NEAT1 were as follows: GGGAAAGCTGTTGGGTTGTA (forward) and GCCTTCCCACTGTTAAACCA (reverse). The primer sequences (5′–3′) for the reference gene glyceraldehyde 3-phosphate dehydrogenase were GGCCTCCAAGGAGTAAGAAA (forward) and GCCCCTCCTGTTATTATGG (reverse).

### Enzyme-linked immunosorbent assay (ELISA)

Culture media were collected and centrifuged at 1000×*g* for 20 min to obtain the supernatant for ELISA. The levels of IL-1β, TNF-α, Arg-1, IL-4, and IL-10 were determined using the Mouse IL-1β ELISA Kit (product number: KS10929), Mouse TNF-α ELISA Kit (product number: KS10484), Mouse Arg-1 ELISA Kit (product number: KS13668), Mouse IL-4 ELISA Kit (product number: KS11806), and Mouse IL-10 ELISA Kit (product number: KS10138), respectively. The five kits were manufactured by Shanghai Keshun Science and Technology Co., Ltd. (China). The levels of IL-6 were measured using a Mouse IL-6 ELISA Kit (product number: L20268-48 T; Shanghai Jianglai Biotechnology Co., Ltd., China) according to the manufacturer’s protocol.

### Immunofluorescence

The cell slides were washed twice with 1 × phosphate-buffered saline (PBS; pH 7.4) and fixed with 4% formaldehyde solution at room temperature for 10 min. After washing the cells thrice with PBS, they were permeabilized with 0.3% Triton X-100 solution at room temperature for 10 min. After washing the cells three times with PBS, a 5% bovine serum albumin-blocking solution was added, and the cells were incubated at room temperature for 15 min. After removing the excess solution without washing, the primary antibody (dilution ratio: 1:50 for CD86 and 1:75 for CD163) was added and incubated at 4 °C overnight. The cells were washed thrice with PBS for 2 min each. Secondary antibodies were diluted 1:500, and Alexa Fluor^®^ 488 (green) goat anti-rabbit IgG (H + L) was added and incubated at room temperature in the dark for 1 h. The cells were washed thrice with PBS for 2 min each. Subsequently, 50 μL of 4,6-diamino-2-phenyl indole (DAPI) staining was added for 10 min. The cells were washed thrice with PBS for 2 min each and mounted with an antiquenching agent. Images were obtained using a fluorescence microscope, and photographs were taken.

### Cell counting kit-8 assay

mCAECs (600 μL, 2 × 10^5^ cells/mL) were seeded in the lower chamber of a transwell cell culture plate. The following day, BV-2 cells from each group (200 μL, 2 × 10^5^ cells/mL) were seeded into the upper chamber of a transwell cell culture plate. After co-culturing for 24 h, the mCAECs were harvested and transferred to a 96-well culture plate. mCAECs were cultured for 0, 3, 5, and 7 days, after which 10 μL of Cell Counting Kit-8 solution was added to each well. Following a 2-h incubation in the cell culture incubator, the absorbance at 450 nm was measured using a microplate reader. The optical density at 450 nm (OD450) of the blank group on day 0 was designated as the control group OD450 nm, whereas the OD450 values of the other groups at various time points were designated as the experimental group OD450 nm. The proliferation rate of mCAECs was calculated using the following formula: cell proliferation rate = (experimental group OD450 nm/control group OD450 nm − 1) × 100%.

### Transwell assay and transwell-matrigel assay

To investigate the effect of different groups of BV-2 cells on the migration ability of mCAECs, a transwell assay was performed. mCAECs (200 μL, 2 × 10^5^ cells/mL) were resuspended in FBS-free culture medium and seeded in the upper chamber of a transwell cell culture plate. BV-2 cells (600 μL, 2 × 10^5^ cells/mL) were resuspended in culture medium containing 20% FBS and seeded into the lower chamber of a transwell cell culture plate. The transwell plates were placed in a cell culture incubator for normal cultivation. After 24 h, the mCAECs that had not migrated through the polycarbonate membrane in the upper chamber were gently removed using a cotton swab. Transwell chambers were fixed in 4% paraformaldehyde solution for 20 min, sequentially rinsed in PBS, stained with crystal violet for 10 min, rinsed again in PBS, and observed under a microscope to count the number of mCAECs that migrated through the polycarbonate membrane in each field. Eight fields were randomly selected for imaging. ImageJ Pro software was used to count the migrated cells in the transwell plate, and the average number of cells in each field was calculated as the number of migrated mCAECs in each group. To investigate the effects of different groups of BV-2 cells on the invasive ability of mCAECs, a transwell-Matrigel assay was performed. In addition to the upper chamber of the transwell cell culture plate pre-coated with Matrigel, the other protocol was the same as that for the transwell assay.

### Tube formation assay

The Matrigel was dissolved overnight at 4 °C and centrifuged for 1 min at 4 °C after complete dissolution. The mixture was gently mixed, and 150 μL of Matrigel was added to the lower chamber of the pre-chilled transwell cell culture plates and incubated at 37 °C for 45 min. Simultaneously, 600 μL of mCAECs (1 × 10^5^ cells/mL) was seeded in the lower chamber and incubated under normal conditions. The next day, 200 μL BV-2 cells (2 × 10^5^ cells/mL) from different groups was seeded into the upper chamber of the transwell cell culture plate. Tube formation was observed after co-culturing for 6 h. Subsequently, six random fields were photographed, and ImageJ Pro software was used to quantify the number of tubes formed, providing an evaluation index of the tube formation ability of mCAECs.

### Western blotting

After various treatments, BV-2 cells or mCAECs from each group were collected, and their total proteins were extracted using RIPA lysis buffer. Subsequently, 30 µg of protein was separated using sodium dodecyl-sulfate polyacrylamide gel electrophoresis and transferred onto a polyvinylidene fluoride (PVDF) membrane. The PVDF membrane was blocked using 5% skim milk powder solution diluted in Tris-buffered saline (TBS), and the primary antibodies (1:1000 for vascular endothelial growth factor receptor 2 (VEGFR2), 1:1000 for nuclear factor kappa B (NF-κB) p65, 1:1000 for phosphorylated (p)-NF-κB p65, 1:2000 for AMPK alpha, 1:2000 for AMPK gamma, and 1:3000 for glyceraldehyde 3-phosphate dehydrogenase) were diluted with 5% skim milk powder solution to incubate the PVDF membrane. The PVDF membrane was washed with TBS containing 1‰ Tween-20 (TBST), and the horseradish peroxidase-conjugated secondary antibody (1:5000 dilution) was diluted with a 5% skim milk powder solution for further incubation of the PVDF membrane. After an additional wash with TBST, the horseradish peroxidase signal was visualized using SuperSignal West Pico PLUS (Thermo Scientific, Rockford, IL, USA), and the signals were captured on an X-ray film.

### Label-free quantification proteomics

BV-2 cells transfected with the pcDNA3.1 + and pcDNA-NEAT1 vectors were collected for label-free proteomic quantification. All label-free quantification proteomic procedures were performed by Wuhan GeneCreate Biological Engineering Co., Ltd. (China). Briefly, total protein was extracted using RIPA lysis buffer. Mass spectrometry was performed after protein denaturation and trypsin digestion. Raw mass spectrometry data were analyzed using MaxQuant (V1.6.6) software with the Andromeda algorithm. Protein identification was performed using the UniProt mouse proteome reference database. The results were filtered at a 1% false discovery rate at both protein and peptide levels, removing reverse and contaminant proteins and proteins with only one modified peptide. The remaining information was used for subsequent statistical, clustering, and differential protein abundance analyses to identify proteins that were differentially expressed in BV-2 cells transfected with pcDNA-NEAT1. Furthermore, all identified differentially expressed proteins were subjected to Gene Ontology terms (including Biological Process, Cellular Component, and Molecular Function) and Kyoto Encyclopedia of Genes and Genomes pathway annotation using the Diamond program in the eggNOG-mapper software, followed by functional enrichment analysis using hypergeometric testing.

### Statistical analysis

Statistical analyses were performed using GraphPad Prism version 7.0 (GraphPad Software, San Diego, CA, USA). One-way analysis of variance (ANOVA) followed by Tukey's multiple comparison test was used to assess the significance of the differences. Statistical significance was set at *P* < 0.05.

## Results

### Effect of NEAT1 on BV-2 polarization induced by LPS

**Fig. 1:**
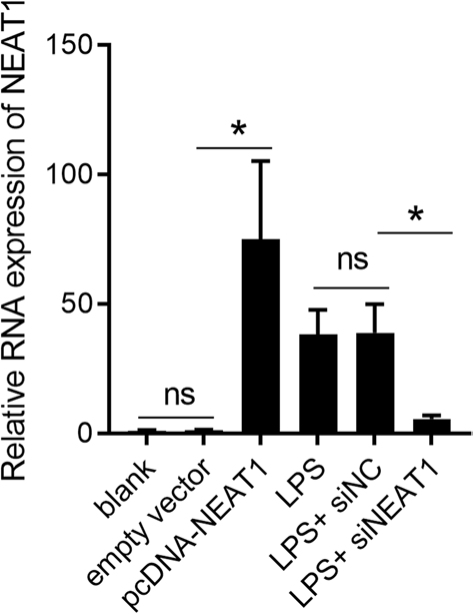
Relative expression of NEAT1 in BV-2 cells of the six groups. ns indicates no statistical difference, *P* > 0.05; *indicates statistically significant difference, *P* < 0.05

**Fig. 2:**
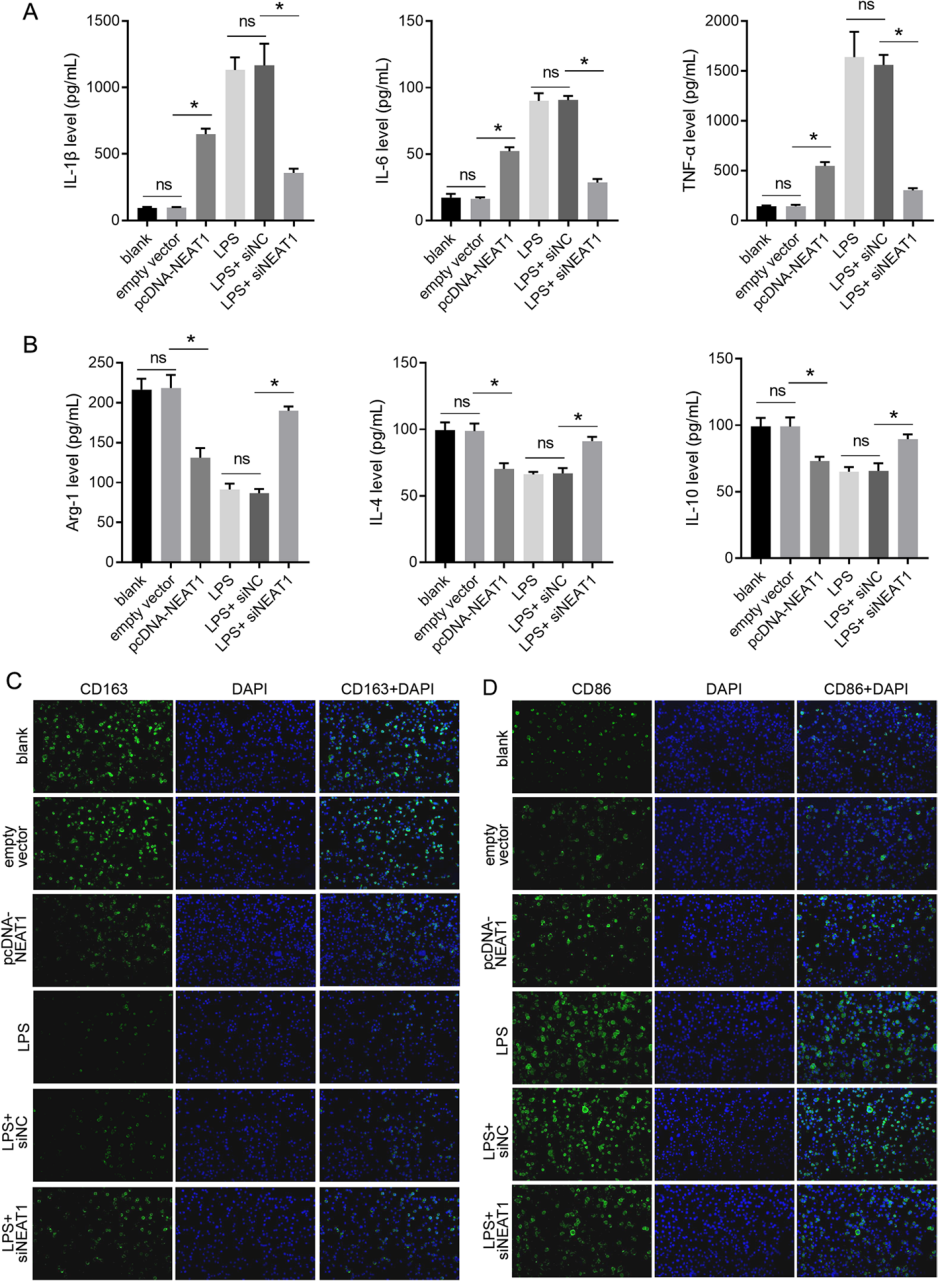
Effect of NEAT1 on the secretion of proinflammatory and anti-inflammatory cytokines and expression level of CD163 and CD86. **A** NEAT1 overexpression and LPS treatment promote the secretion of proinflammatory cytokines L-1β, IL-6, and TNF-α, while NEAT1 knockdown alleviates the effect of LPS. **B** NEAT1 overexpression and LPS treatment suppresses the secretion of anti-inflammatory cytokines Arg-1, IL-4, and IL-10, while NEAT1 knockdown alleviates the effect of LPS. **C** NEAT1 overexpression and LPS treatment decrease the rate of CD163-positive BV-2 cells, while NEAT1 knockdown alleviates the effect of LPS. **D** NEAT1 overexpression and LPS treatment increase the rate of CD86-positive BV-2 cells, while NEAT1 knockdown alleviates the effect of LPS. ns indicates no statistical difference, *P* > 0.05; *indicates statistically significant difference, *P* < 0.05

To investigate the role of NEAT1 in microglial polarization, NEAT1 was overexpressed in BV-2 cells and knocked down in LPS-stimulated BV-2 cells (Fig. 1). Next, we examined the ability of different BV-2 cell lines to secrete proinflammatory and anti-inflammatory cytokines. The levels of the proinflammatory cytokines IL-1β, IL-6, and TNF-α were enhanced, whereas the levels of the anti-inflammatory cytokines Arg-1, IL-4, and IL-10 were lower in the pcDNA-NEAT1 group than in the empty vector group (Fig. 2A, B). Compared with the LPS + siNC group, the levels of IL-1β, IL-6, and TNF-α in the LPS + siNEAT1 group were lower, whereas the levels of Arg-1, IL-4, and IL-10 were higher (Fig. 2A, B). Furthermore, we analyzed the expression levels of CD163 and CD86. The results showed that the expression level of CD163 decreased, whereas the expression level of CD86 increased in the pcDNA-NEAT1 group compared with the empty vector group (Fig. 2C, D). The expression of CD163 was higher in the LPS + siNEAT1 group than that in the LPS + siNC group, whereas that of CD86 was lower (Fig. 2C, D).

### Effect of NEAT1-overexpressing or -knocking down BV-2 cell on mCAECs

**Fig. 3:**
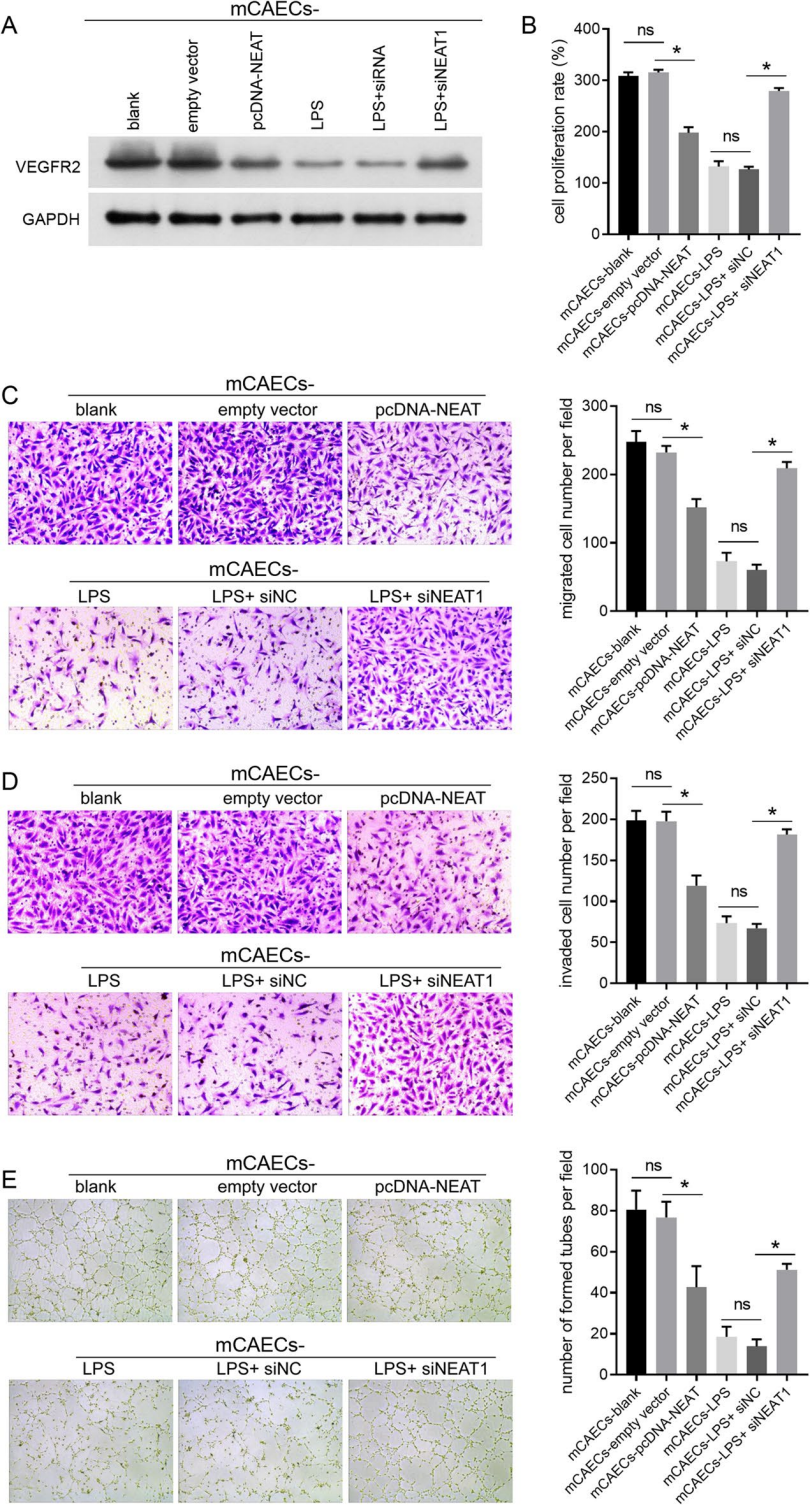
Effect of NEAT1-overexpressing or NEAT1-knocking down BV-2 cells on the VEGFR2 expression, cell proliferation, cell migration, cell invasion, and tube-forming abilities of mCAECs. mCAECs were co-cultured with BV-2 cells in different groups using a transwell system. VEGFR2 expression (**A**), cell proliferation (**B**), cell migration (**C**), cell invasion (**D**), and tube-forming abilities of mCAECs were suppressed by BV-2 cells overexpressing NEAT1, while enhanced by BV-2 cells knocking down NEAT1 under LPS treatment. ns indicates no statistical difference, *P* > 0.05; *indicates a statistically significant difference, *P* < 0.05

To determine whether the overexpression or knockdown of NEAT1 in BV-2 cells affects the angiogenic capability of mCAECs, we measured the effect of different groups of BV-2 cells on VEGFR2 expression, cell proliferation, cell migration, cell invasion, and the tube-forming ability of mCAECs using a transwell co-culture system. The VEGFR2 expression level, cell proliferation rate, number of migrated and invaded mCAECs, and number of tubes formed by mCAECs were lower in the mCAECs-pcDNA-NEAT1 group than in the mCAEC-empty vector group (Fig. 3A–E). Moreover, the VEGFR2 expression level, cell proliferation rate, number of migrated and invaded mCAECs, and number of tubes formed by mCAECs in the mCAECs-LPS + siNEAT1 group were higher than those in the mCAECs-LPS + siNC group (Fig. 3A–E).

### Differentially expressed proteins induced by NEAT1 overexpression

**Fig. 4:**
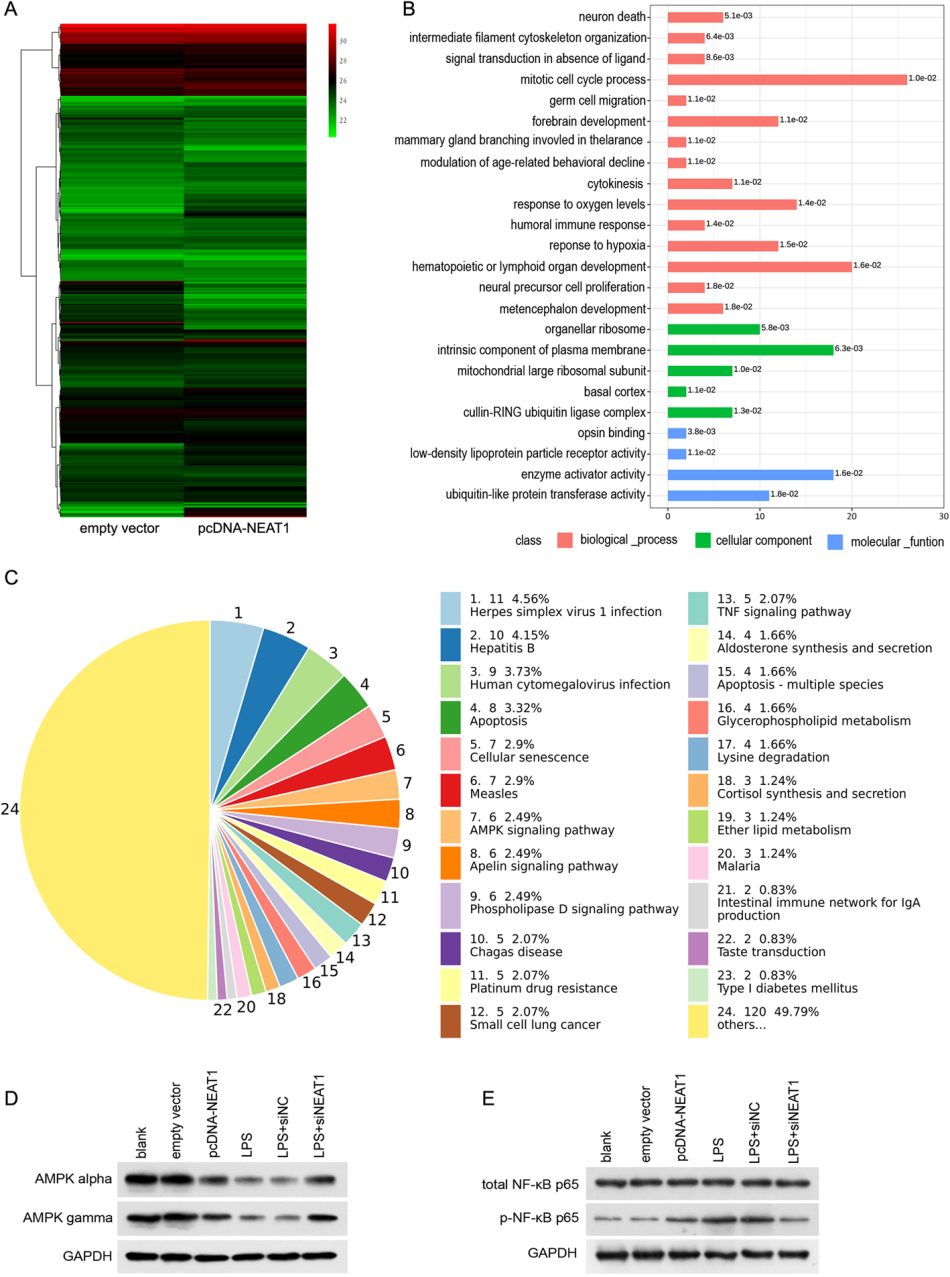
The AMPK signaling pathway was enriched from differentially expressed proteins in NEAT1-overexpressing BV-2 cells identified using label-free quantification proteomics. **A** Hierarchical clustering analysis of all identified differentially expressed proteins. **B** GO term enrichment of all identified differentially expressed proteins. **C** KEGG pathway enrichment of all identified differentially expressed proteins. **D**, **E** Effect of NEAT1 on the expression levels of AMPK alpha, AMPK gamma, total NF-κB p65, and p-NF-κB p65 in BV-2 cells. *KEGG* Kyoto Encyclopedia of Genes and Genomes, *GO* Gene Ontology

To explore the mechanism through which NEAT1 regulates microglial polarization and angiogenesis in mCAECs, we identified differentially expressed proteins in NEAT1-overexpressing BV-2 cells using label-free quantification proteomics. Compared with the empty vector group, 144 upregulated and 131 downregulated proteins were identified in the pcDNA-NEAT1 group of BV-2 cells. Information on all identified differentially expressed proteins is shown in Additional file 1: Table S1, and hierarchical clustering analysis of all identified differentially expressed proteins is shown in Fig. 4A. The top 20 upregulated and downregulated proteins are listed in Tables 1 and 2, respectively.

**Table 1:** Information of top 20 up-regulated proteins

Accession	Gene	Description	Fold change (log2)
O70494	Sp3	Transcription factor Sp3	6.99
Q6ZWY3	Rps27l	40S ribosomal protein S27-like	6.29
Q6ZWY8	Tmsb10	Thymosin beta-10	5.62
P62073	Timm10	translocase of inner mitochondrial membrane 10	5.20
P97411	Ica1	Islet cell autoantigen 1	4.86
Q8K1A5	Tmem41b	Transmembrane protein 41B	4.58
Q8VHL1	Setd7	Histone-lysine N-methyltransferase SETD7	4.29
Q8VDV8	Mitd1	MIT domain-containing protein 1	4.09
Q08857	Cd36	Platelet glycoprotein 4	4.05
Q09143	Slc7a1	High affinity cationic amino acid transporter 1	3.83
Q8BP40	Acp6	Lysophosphatidic acid phosphatase type 6	3.79
Q69ZQ2	Isy1	Pre-mRNA-splicing factor ISY1 homolog	3.48
Q9ET26	Rnf114	E3 ubiquitin-protein ligase RNF114	3.35
Q3U1Y4	Dennd4b	DENN domain-containing protein 4B	3.33
Q91WE2	Psme3ip1	PSME3-interacting protein	3.29
Q9JMD0	Znf207	BUB3-interacting and GLEBS motif-containing protein ZNF207	3.19
O08856	Ell	RNA polymerase II elongation factor ELL	3.12
P51908	Apobec1	apolipoprotein B mRNA editing enzyme, catalytic polypeptide 1	2.99
Q80WT5	Aftph	Aftiphilin	2.89
P26323	Fli1	Friend leukemia integration 1 transcription factor	2.82

**Table 2:** Information of top 20 down-regulated proteins

Accession	Gene	Description	Fold change (log2)
Q8R0W0	Eppk1	Epiplakin	− 6.09
Q91VR8	Brk1	Protein BRICK1	− 5.41
Q9JL62	Gltp	Glycolipid transfer protein	− 4.43
P59279	Rab2b	Ras-related protein Rab-2B	− 4.16
P43277	H1-3	Histone H1.3	− 3.98
Q9CR61	Ndufb7	NADH dehydrogenase [ubiquinone] 1 beta subcomplex subunit 7	− 3.53
P61963	Dcaf7	DDB1- and CUL4-associated factor 7	− 2.85
Q6P5C5	Smug1	Single-strand selective monofunctional uracil DNA glycosylase	− 2.85
Q9CQC8	Spg21	Maspardin	− 2.83
Q61025	Ift20	Intraflagellar transport protein 20 homolog	− 2.82
Q5SVR0	Tbc1d9b	TBC1 domain family member 9B	− 2.79
Q6P8M1	Tatdn1	Deoxyribonuclease TATDN1	− 2.71
O35955	Psmb10	Proteasome subunit beta type-10	− 2.65
Q9DBL2	Gdap2	Ganglioside-induced differentiation-associated protein 2	− 2.60
Q14CH7	Aars2	Alanine–tRNA ligase, mitochondrial	− 2.60
Q91VT4	Cbr4	3-Oxoacyl-[acyl-carrier-protein] reductase	− 2.60
Q8K4R9	Dlgap5	Disks large-associated protein 5	− 2.47
Q8CC86	Naprt	Nicotinate phosphoribosyltransferase	− 2.43
Q8K2F8	Lsm14a	Protein LSM14 homolog A	− 2.41
Q5SRY7	Fbxw11	F-box/WD repeat-containing protein 11	− 2.36

To further analyze the molecular mechanisms by which NEAT1 exerts its function, differentially expressed proteins were subjected to Gene Ontology analysis to determine the biological processes, molecular functions, and cellular locations in which they are involved. As shown in Fig. 4B, the biological processes in which differentially expressed proteins participate include the mitotic cell cycle, hematopoietic or lymphoid organ development, and response to oxygen levels. The molecular functions of the differentially expressed proteins include enzyme activator activity, ubiquitin-like protein transferase activity, opsin binding, and low-density lipoprotein particle receptor activity. The cellular locations of the differentially expressed proteins include the intrinsic components of the plasma membrane, organellar ribosome, cullin-RING ubiquitin ligase complex, mitochondrial large ribosomal subunit, and basal cortex.

To further analyze the downstream signaling pathways regulated by NEAT1, differentially expressed proteins were subjected to the Kyoto Encyclopedia of Genes and Genomes analysis. As shown in Fig. 4C, the AMPK and TNF signaling pathways were enriched.

### Effect of NEAT1 on AMPK and NF-κB signaling pathways in BV-2 cells

To validate the proteomics results, changes in the expression of key proteins in the AMPK signaling pathway and its downstream NF-κB signaling pathway were analyzed. The expression levels of AMPK catalytic subunit alpha and regulatory subunit gamma were lower in the pcDNA-NEAT1 group than in the empty vector group and higher in the LPS + siNEAT1 group than in the LPS + siNC group (Fig. 4D). The expression levels of p-NF-κB p65 were higher in the pcDNA-NEAT1 group than in the empty vector group and lower in the LPS + siNEAT1 group than in the LPS + siNC group (Fig. 4E). The expression level of total NF-κB p65 did not change in any of the groups.

### AMPK activator AICAR reversed the effect of NEAT1 on BV-2 polarization

**Fig. 5:**
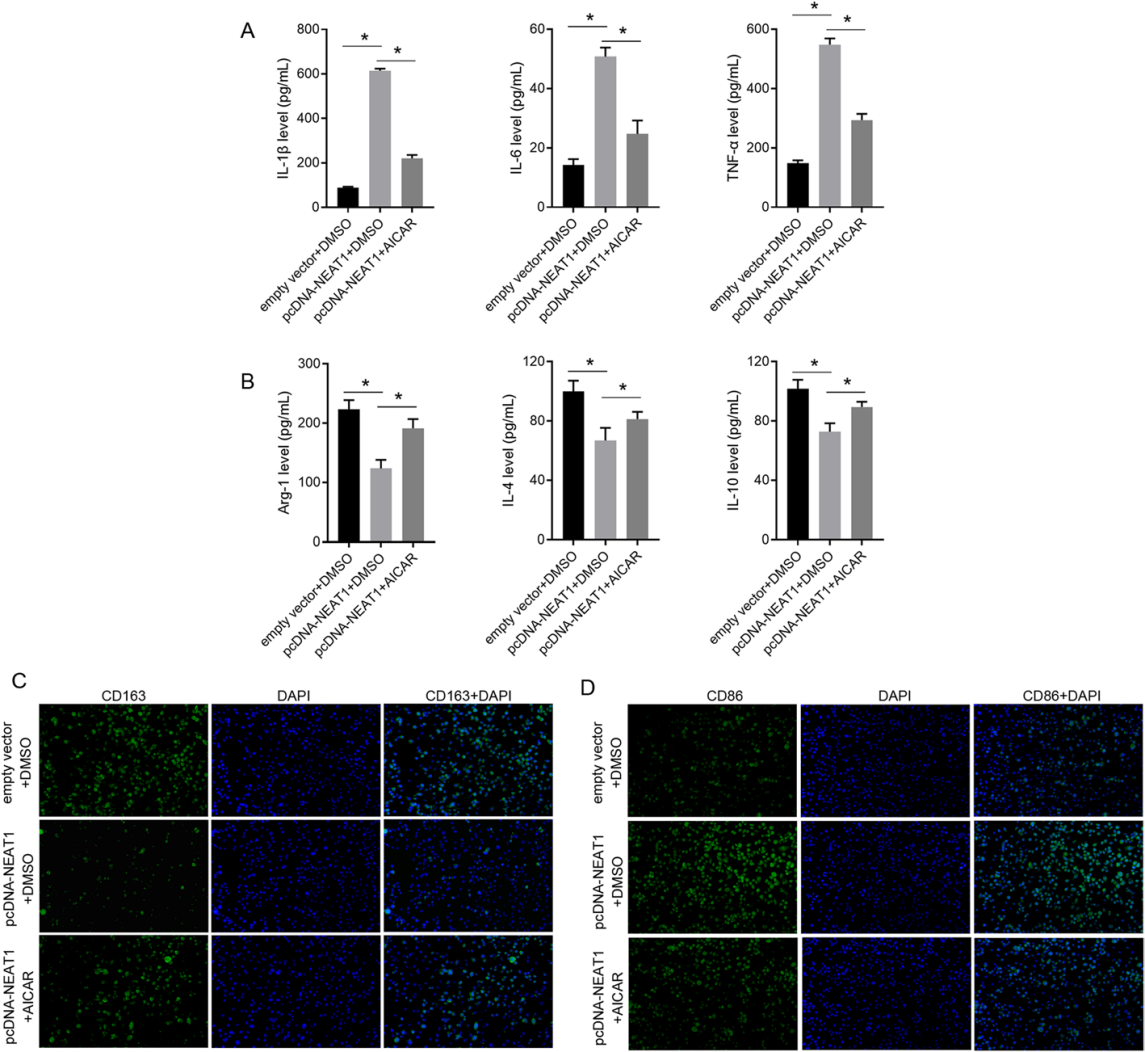
AMPK activator AICAR reversed the effect of NEAT1 on BV-2 polarization. In NEAT1-overexpressing BV-2 cells (transfected with NEAT1-overexpressing plasmid pcDNA-NEAT1), AICAR suppressed the secretion of proinflammatory cytokines L-1β, IL-6, and TNF-α (**A**), promoted the secretion of anti-inflammatory cytokines Arg-1, IL-4, and IL-10 (**B**), increase the rate of CD163-positive BV-2 cells (**C**), and decrease the rate of CD86-positive BV-2 cells (**D**). *Indicates a statistically significant difference, *P* < 0.05

To further confirm whether NEAT1 regulates BV-2 polarization through the AMPK signaling pathway, the AMPK activator AICAR was used to treat NEAT1-overexpressing BV-2 cells. Compared with the pcDNA-NEAT1 + DMSO group, the levels of IL-1β, IL-6, and TNF-α in the pcDNA-NEAT1 + AICAR group were lower, whereas the levels of Arg-1, IL-4, and IL-10 were higher (Fig. 5A, B). The expression of CD163 was higher in the pcDNA-NEAT1 + AICAR group than that in the pcDNA-NEAT1 + DMSO group, whereas that of CD86 was lower (Fig. 5C, D). These results showed that the AMPK activator AICAR reversed the effect of NEAT1 on BV-2 polarization.

### AMPK activator AICAR reversed the regulatory effect of NEAT1 on mCAECs

**Fig. 6:**
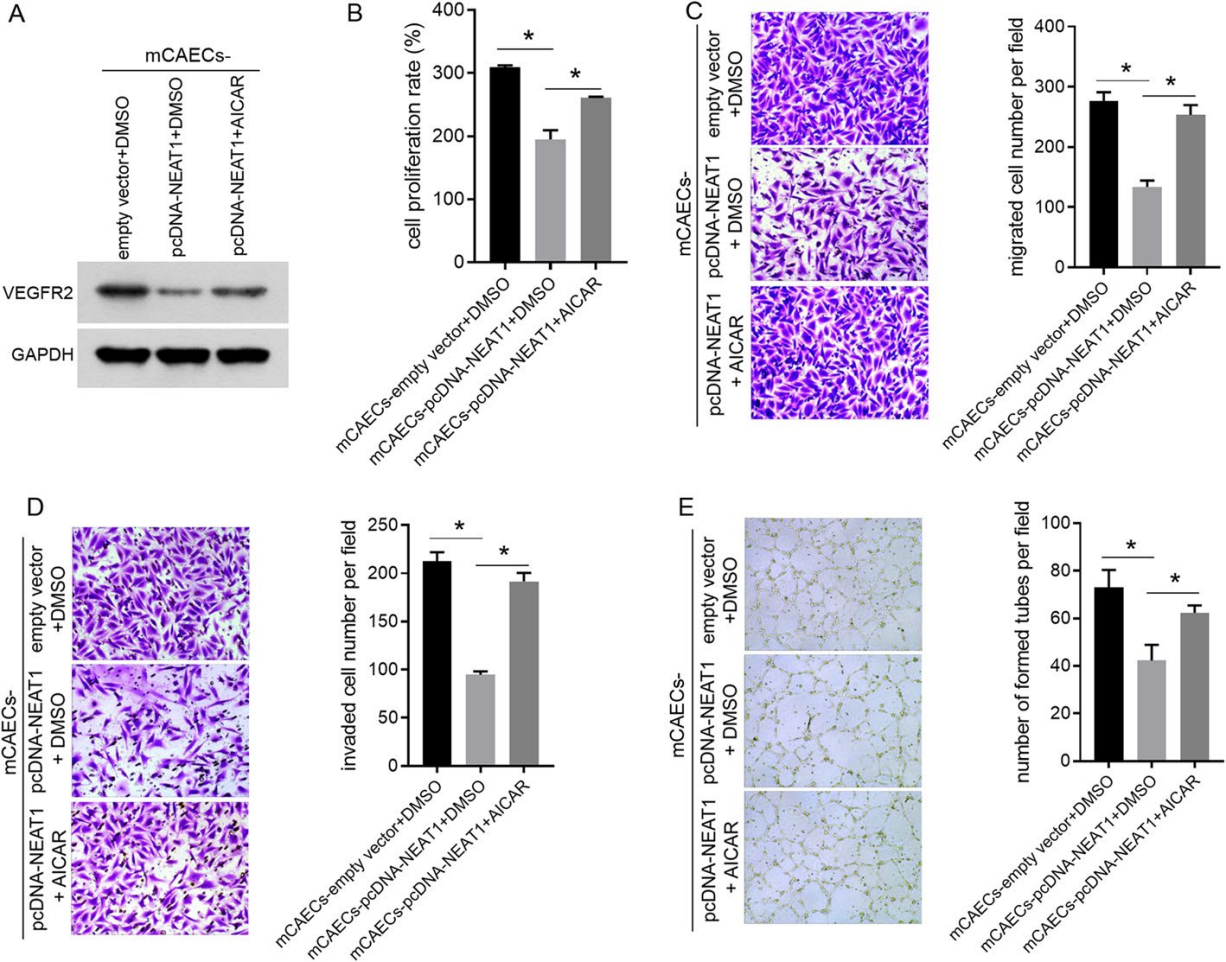
AMPK activator AICAR reversed the regulatory effect of NEAT1-overexpressing BV-2 cells on the VEGFR2 expression, cell proliferation, cell migration, cell invasion, and tube-forming abilities of mCAECs. mCAECs were co-cultured with BV-2 cells in different groups using a transwell system. VEGFR2 expression (**A**), cell proliferation (**B**), cell migration (**C**), cell invasion (**D**), and tube-forming abilities of mCAECs were enhanced by AMPK activator AICAR. *Indicates a statistically significant difference, *P* < 0.05

To further confirm whether NEAT1 regulates the angiogenic ability of mCAECs through the AMPK signaling pathway, the AMPK activator AICAR was used to treat NEAT1-overexpressing BV-2 cells to reverse the effect of NEAT1 on the AMPK signaling pathway. The effects of this treatment on the proliferation, migration, invasion, tube formation, and VEGFR2 expression in mCAECs were analyzed. The VEGFR2 expression level, cell proliferation rate, the number of migrated and invaded mCAECs, and the number of tubes formed by mCAECs were increased in mCAECs-pcDNA-NEAT1 + AICAR compared with mCAECs-pcDNA-NEAT1 + DMSO (Fig. 6A–E). These results showed that the AMPK activator AICAR reversed the regulatory effect of NEAT1-overexpressing BV-2 cells on mCAECs.

## Discussion

For the first time, this study explored the impact of changes in NEAT1 expression in microglial cells on the angiogenic ability of cerebrovascular endothelial cells and the associated molecular mechanisms using proteomic methods. BV-2 is an immortalized cell line commonly used to study microglial function in mice [23]. The LPS-induced BV-2 cell model is commonly employed in studies of microglial polarization [24, 25]. Therefore, this model was used in the present study.

The present study showed that NEAT1 overexpression can enhance proinflammatory cytokine secretion and the CD86-positive rate while decreasing anti-inflammatory cytokine secretion and the CD163-positive rate in BV-2 cells. CD86 and CD163 are the surface markers of M1 and M2 microglia, respectively. These results suggest that NEAT1 induces M1 polarization in microglia. The effect of NEAT1 is similar to that of LPS [24, 25]. Moreover, NEAT1 knockdown decreases proinflammatory cytokine secretion and the CD86-positive rate while increasing anti-inflammatory cytokine secretion and the CD163-positive rate in LPS-treated BV-2 cells. These results demonstrate that NEAT1 knockdown blocks LPS-induced M1 microglial polarization. The role of NEAT1 in regulating M1 polarization also been studied by other researchers. In oxygen–glucose deprivation/reoxygenation exposed BV-2 cells, NEAT1 knockdown decreased the mRNA levels of M1 markers, suggesting that NEAT1 knockdown suppresses M1 microglial polarization [21]. In titanium particle-stimulated mouse bone marrow derived macrophages (BMDMs), NEAT1 knockdown reduced the proportion of M1 polarization [26]. In RAW264.7 cells and mouse BMDMs, NEAT1 overexpression could induce the differentiation into M1 type [27]. NEAT1 showed the same regulatory effect on M1 polarization in different cell models. All these studies suggest that NEAT1 may be a critical target for controlling microglial phenotypic transformations.

Our present study showed that NEAT1 inactivated the AMPK signaling pathway and NEAT1 knockdown abolished the inhibitory effect of LPS on AMPK signaling pathway in BV-2 cells. Previous study showed that NEAT1 knockdown activated AMPK signaling pathway in free fatty acids-treated HepG2 cells, a cell model of nonalcoholic fatty liver disease [28]. All these results suggest that NEAT1 may play its role through AMPK signaling pathway. Furthermore, we found that AMPK activator AICAR reversed the effect of NEAT1 on BV-2 polarization. It is known that activating the AMPK signaling pathway can drive microglia toward the M2 phenotype [29, 30]. Therefore, our present study suggests that NEAT1 plays its role in microglial polarization through AMPK signaling pathway.

Moreover, we found that NEAT1 could activate the NF-κB signaling pathway, which is downstream of the AMPK signaling pathway. AMPK inhibits the NF-κB signaling pathway through downstream target molecules such as sirtuin 1 [31]. The NF-κB pathway is widely recognized as a central signaling pathway in macrophage phenotypic transition [32]. The core protein of the phosphorylated NF-κB signaling pathway is NF-κB p65, and phosphorylation of this protein is a characteristic feature of NF-κB signaling pathway activation [32]. Based on the above, we hypothesized that NEAT1 may play a role in microglial polarization through the AMPK/NF-κB signaling pathway. However, this hypothesis needs further experiments to verify.

Angiogenesis involves various cell types, including endothelial and vascular smooth muscle cells. When studying this process in the laboratory, researchers commonly use the growth, movement, invasion, and tube-forming abilities of endothelial cells as indicators of their capacity to create new blood vessels [33–35]. Several studies have shown that the stronger these abilities, the better the cells are at regenerating blood vessels [33–35]. VEGF plays a key role in regulating this process, making it a primary candidate for gene therapy in ischemic stroke [36, 37]. VEGF has three receptors: VEGFR1, VEGFR2, and VEGFR3, among which VEGFR2 produces signals for generating blood vessels [36, 37]. In the present study, we found that NEAT1-overexpressing BV-2 cells exhibited decreased VEGFR2 expression and alleviated the proliferation, migration, invasion, and tube-forming abilities of mCAECs. Moreover, NEAT1-knocking BV-2 cells alleviated the suppressive effect of LPS on VEGFR2 expression, cell proliferation, cell migration, cell invasion, and the tube-forming abilities of mCAECs. These results reveal that NEAT1-overexpressing BV-2 cells can suppress the angiogenic ability of cerebrovascular endothelial cells and that NEAT1-knocking BV-2 cells can promote the angiogenic ability of cerebrovascular endothelial cells in an inflammatory microenvironment. In addition, our results showed that NEAT1 induced M1 polarization in microglia. Therefore, we hypothesized that NEAT1 participates in the regulation of cerebrovascular regeneration by influencing microglial polarization.

Furthermore, our results showed that the AMPK activator AICAR reversed the regulatory effect of NEAT1-overexpressing BV-2 cells on VEGFR2 expression, cell proliferation, cell migration, cell invasion, and tube-forming abilities of mCAECs. Numerous researchers have demonstrated that the activation of AMPK signaling pathway enhances angiogenesis [38]. In addition, M1 type microglia suppress angiogenesis [39]. Therefore, our results indicate that NEAT1 may inhibit the angiogenic activity of cerebrovascular endothelial cells by inducing the M1 polarization of microglia through the AMPK signaling pathway.

However, our present research has some limitations. Firstly, we did not investigate the effect of NEAT1 knockdown without LPS on M1/M2 polarization of BV-2 cells, AMPK/NF-κB signaling pathway in BV-2 cells, and the angiogenic ability of mCAECs. It is not entirely convincing to infer the effect of NEAT1 knockdown from the effect of NEAT1 overexpression. Secondly, the current results do not fully explain how NEAT1 regulates the expression of AMPK alpha and gamma proteins. Further experiments are required to analyze the regulatory role of NEAT1 in its interaction with AMPK. Finally, there is a lack of animal models to validate the effects of NEAT1 on microglial polarization and angiogenesis.

## Conclusions

The present study showed that NEAT1 inhibits the angiogenic activity of mCAECs by inducing M1 polarization of BV-2 cells through the AMPK signaling pathway. This study further clarified the impact of NEAT1 on microglia, enriching research in this field. We also explored the role of NEAT1 in the regulation of microglial polarization. This study fills several gaps in our knowledge regarding the relationship between NEAT1 and microglia and provides a solid experimental foundation for future research.

### Supplementary Information

**Additional file 1. Table S1:** The differentially expressed proteins between the pcDNA-NEAT1 and empty vector groups were shown.

## Data Availability

All data from this study are available from the corresponding author.

## Abbreviations

NEAT1: Nuclear paraspeckle assembly transcript 1
mCAECs: Mouse cerebral arterial endothelial cells
AMPK: AMP-activated protein kinase
IL: Interleukin
TNF-α: Tumor necrosis factor-α
Arg-1: Arginase-1
FBS: Fetal bovine serum
siNC: Negative control siRNA
LPS: Lipopolysaccharide
siNEAT1: SiRNA targeting NEAT1
DMSO: Dimethyl sulfoxide
AICAR: 5-Aminoimidazole-4-carboxamide ribonucleotide
PCR: Polymerase chain reaction
ELISA: Enzyme-linked immunosorbent assay
PBS: Phosphate-buffered saline
DAPI: 4,6-Diamino-2-phenyl indole
OD450: Optical density at 450 nm
TBS: Tris-buffered saline
PVDF: Polyvinylidene fluoride
VEGFR2: Vascular endothelial growth factor receptor 2
NF-κB: Nuclear factor kappa B
p-NF-κB p65: Phosphorylated-NF-κB p65
TBST: TBS containing 1‰ Tween-20
BMDMs: Bone marrow derived macrophages
